# Diagnostic accuracy of point-of-care urine tenofovir test, and associations between metrics of tenofovir use and treatment outcomes in a community ART programme

**DOI:** 10.64898/2026.08.13.26360358

**Published:** 2026-08-14

**Authors:** Lisanthini Naidu, Kwena Tlhaku, Katya Govender, Yukteshwar Sookrajh, Pravikrishnen Moodley, Johan van der Molen, Natasha Samsunder, Lara Lewis, Monica Gandhi, Paul K. Drain, Christopher C Butler, Gail Hayward, Nigel Garrett, Jienchi Dorward

## Abstract

**Background:**

Urine tenofovir (uTFV) and dried blood spot (DBS) tenofovir diphosphate (TFV-DP) concentrations respectively estimate short- and medium-term adherence to tenofovir disoproxil fumarate (TDF)-based antiretroviral therapy (ART). We evaluated the accuracy of a point-of-care uTFV assay, and associations between uTFV/TFV-DP, and viral load (VL) and retention outcomes within a South African community ART programme.

**Methods:**

We measured uTFV and DBS TFV-DP concentrations using liquid chromatography-tandem mass spectrometry (LC-MS/MS). We calculated sensitivity and specificity of the point-of-care uTFV assay at the manufacturer-recommended threshold of ≥1,500 ng/mL compared to LC-MS/MS. We assessed associations of the point-of-care uTFV assay, and DBS TFV-DP concentrations with concurrent viraemia, and with retention-in-care by 16 weeks post-enrolment.

**Results:**

Of 196 adults median age was 44 years, 127 (64.8%) were female, and 191 (97.4%) were receiving TDF. 185 (94.4%) had detectable point-of-care uTFV, which had high sensitivity (99.5%, 95% CI 96.5-100%) and moderate specificity (76.9%, 95% CI 46.0-93.8%) for detecting uTFV ≥1,500 ng/mL. Two participants had concurrent viraemia ≥1,000 copies/mL; of these 50.0% (95% CI 9.4-90.5) had undetectable point-of-care uTFV, and 100% (95% CI 19.7-100) had low TFV-DP <483 fmol/punch. Among participants without viraemia 97.4% (95% CI 93.6-99.0) had detectable uTFV, and 98.1% (95.3-99.6) had high TFV-DP ≥483 fmol/punch. Point-of-care uTFV and DBS TFV-DP were not associated with retention-in-care.

**Conclusions:**

The point-of-care uTFV assay demonstrated high sensitivity and moderate specificity to detect uTFV. Over 95% of people without viraemia had detectable point-of-care uTFV or high DBS TFV-DP levels respectively, but these were not associated with 16-week retention-in-care.

## Full Text Availability

The license terms selected by the author(s) for this preprint version do not permit archiving in PMC. The full text is available from the preprint server.

